# Production Inhibition and Excretion Promotion of Urate by Fucoidan from *Laminaria japonica* in Adenine-Induced Hyperuricemic Mice

**DOI:** 10.3390/md16120472

**Published:** 2018-11-27

**Authors:** Dayan Zhang, Huazhong Liu, Ping Luo, Yanqun Li

**Affiliations:** 1 College of Chemistry & Environment, Guangdong Ocean University, Zhanjiang 524088, China; 902030@sina.com or a15810529648@163.com (D.Z.); luopingna@163.com (P.L.); 2 College of Food Science & Technology, Guangdong Ocean University, Zhanjiang 524088, China

**Keywords:** fucoidan, hepatorenal functions, urate, mice

## Abstract

This work aims to explore the amelioration of fucoidan on adenine-induced hyperuricemia and hepatorental damage. Adenine-induced hyperuricemic mice were administered with fucoidan, allopurinol and vehicle control respectively to compare the effects of the drugs. Serum uric acid, urea nitrogen, hepatorenal functions, activities of hepatic adenosine deaminase (ADA), xanthine oxidase (XOD), renal urate transporter 1 (URAT1) and NF-κB p65 were assessed. As the serum uric acid, urea nitrogen, creatinine, glutamic oxalacetic transaminase (GOT), glutamic pyruvic transaminase (GPT), superoxide dismutase (SOD), catalase (CAT) and malondialdehyde (MDA) data demonstrated, the adenine not only mediated hepatorenal function disorders, but also induced hyperuricemia in mice. Meanwhile, activities of hepatic ADA and XOD were markedly augmented by adenine, and the expression of URAT1 was promoted, which was conducive to the reabsorption of urate. However, exposure to fucoidan completely reversed those adenine-induced negative alternations in mice, and the activities of hepatic ADA and XOD were recovered to the normal level. It was obvious that hepatic and renal functions were protected by fucoidan treatment. The expression of URAT1 was returned to normal, resulting in an increase of renal urate excretion and consequent healing of adenine-induced hyperuricemia in mice. Expression and activation of NF-κB p65 was promoted in kidneys of adenine treated mice, but suppressed in kidneys of mice exposed to fucoidan from *Laminaria japonica* or allopurinol. In conclusion, the fucoidan is a potential therapeutic agent for the treatment of hyperuricemia through dual regulatory roles on inhibition of hepatic metabolism and promotion of renal excretion of urate.

## 1. Introduction

Hyperuricemia is an imbalance between production and excretion of urate, which can cause many disorders including gout, chronic nephritis, renal dysfunction, cardiovascular diseases, hypertension, diabetes and metabolic syndromes [1,2,3]. It is therefore very important to decrease the serum uric acid (SUA) level for preventing and treating these diseases.

Based on different mechanisms, two types of agent for the treatment of hyperuricemia have been distinguished. Uricostatic drugs, such as allopurinol and febuxostat, are used to repress activities of urate synthesizing enzymes to inhibit the production of uric acid. Another type of agent are uricosuric drugs, including sulphinpyrazone, probenecid and benzbromarone, which can promote renal excretion of uric acid through blocking renal tubular reabsorption of urate [4,5]. It is notable that increasing evidence of the drugs’ side effects have been uncovered [5,6]. Hence, searching for new effective and nontoxic agents to replace the current chemicals is a critical task for the treatment of hyperuricemia. Natural products and Chinese traditional medicines have been considered to be good options [7,8].

Fucoidan is a type of sulfated and heterogeneous polysaccharide, mainly found in the cell-wall matrix of various brown seaweed species, that are rich in fucose and sulfate groups, as well as one or more small proportions of xylose, mannose, galactose, rhamnose, arabinose, glucuronic acid and acetyl groups in different kinds of brown seaweeds [9]. The backbone of fucoidan from *Laminaria japonica* (FL) is primarily (1–3) linked α-l-fucopyranose residues and a few (1–4) linked α-l-fucopyranose. The branch points are at C-4 of 3-linked α-l-fucopyranose residues by β-d-galactopyranose units or at C-2 of 3-linked α-l-fucopyranose residues by non-reducing terminal fucose units. Sulfate groups are linked at the position of C-4 or C-2, sometimes linked to fucose residues at C-4, and to galactose residues at C-3 and/or C-4 [10,11]. A large number of studies have testified that fucoidan possesses diverse functions, including antitumor, antivirus, antioxidation, antithrombotic, anticoagulant, anti-inflammatory, immunomodulatory, as well as effects against various renal, hepatic and uropathic disorders [12,13]. Several reports have testified the protection of fucoidan against renal damage induced by albumin, oxalate and adenine [14,15,16,17]. Li et al. found that FL prevented renal epithelial mesenchymal transition induced by TGF-β1 or FGF-2 [18] and acute kidney injury induced by glycerol [19]. The kidney is an important organ responsible for the excretion of urate, a decisive factor resulting in hyperuricemia and correlative disorders [1,2,20,21], therefore, it is implied by the literature that fucoidan might be a potential therapeutic agent for treatment of hyperuricemia. However, no direct evidence has indicated the urate-reducing effect of the sulfated polysaccharide yet. This paper found that FL effectively decreased serum uric acid level through inhibiting production and promoting renal excretion of urate in hyperuricemic mice induced by adenine.

## 2. Results

### 2.1. Blocking Adenine-Induced Increase of Serum Uric Acid in Mice

As shown in Table 1, adenine succeeded in elevating SUA to a significantly higher content in mice compared to vehicle treatment, which indicated that a hyperuricemia mouse model was established successfully. Meanwhile the similar serum urate levels of vehicle- and allopurinol-treated mice showed that conversion of adenine to uric acid was blocked by allopurinol. Fortunately there was similar SUA content to vehicle- and allopurinol-exposed mice, which was also observed in three groups of fucoidan administered mice. Although no significant difference in SUA content was found among the groups of three doses of fucoidan, vehicle and allopurinol, SUA content in the group of 150 mg/kg fucoidan was slightly lower than that in the groups of other two doses of fucoidan. The data showed that fucoidan efficiently repressed the increase of SUA content in adenine-administered mice, suggesting a potential treatment of fucoidan for hyperuricemia.

### 2.2. Hepatoprotection of Fucoidan in Adenine Administered Mice

Hepatic function was assessed by liver relative weight (LRW), antioxidative indicators in liver and enzyme activities in serum. The apparent effect was shown on alternation of body weight and liver relative weight (Table 2). All mice exposed to adenine grew significantly poorer compared to vehicle treated mice from week 1 to the end. However, no visible difference of body weight was found among all adenine exposed mice, including adenine, adenine plus allopurinol, and adenine plus fucoidan groups. At the end of the trial, LRW was determined. The results showed that though adenine increased the LRW, allopurinol and fucoidan (150 and 200 mg/kg) kept LRW levels at a normal state.

Table 1 presents the activities of both glutamic oxalacetic transaminase (GOT) and glutamic pyruvic transaminase (GPT) in sera of adenine administered mice, which were pronouncedly higher than that of vehicle treated mice. Although GOT activity was successfully reduced to the level of control mice by allopurinol and fucoidan, GPT activity failed to be reduced in allopurinol treated mice, but decreased by fucoidan.

The above-mentioned data suggested that allopurinol and fucoidan prevented mice from the hepatic injury induced by adenine. The prevention effect was also demonstrated by the redox status of liver. The data in Table 3 revealed significant decreases of both superoxide dismutase (SOD) and catalase (CAT) activities, and an increase of malondialdehyde (MDA) content in liver of adenine treated mice compared to control mice. Allopurinol failed to restore activities of both SOD and CAT to normal status, but fucoidan succeeded.

### 2.3. Attenuation of Adenine Mediated Renal Damage by Fucoidan

Renal function was evaluated by kidney relative weight (KRW), the level of antioxidative indicators in kidneys and contents of creatinine and urea nitrogen in serum. Besides allopurinol, fucoidan also reversed adenine mediated augment of KRW in mice (Table 2). Meanwhile, creatinine and urea nitrogen content in sera of mice were augmented markedly by adenine. Although allopurinol, as a therapeutic drug for hyperuricemia treatment, efficiently suppressed adenine induced elevation of creatinine content, it was unable to affect the adenine-caused alteration of urea nitrogen content. Fortunately, fucoidan declined both creatinine and urea nitrogen content (Table 1). Additionally, the SOD and CAT activities were decreased significantly by adenine and were fully recovered by both allopurinol and fucoidan. Moreover, MDA content in the kidneys of adenine treated mice, which was significantly higher than that of control mice, was decreased to normal by fucoidan. Allopurinol clearly reduced MDA content in the kidneys of adenine administered mice, but, as an inhibitor of uric acid metabolism, it was incapable of declining MDA to the level of vehicle treated mice (Table 3).

Based on the above data, including SUA content, hepatic and renal function, the scheduled doses of fucoidan (100, 150 and 200 mg/kg) effectively prevented adenine-induced hyperuricemia and associated disorders. 150 mg/kg of fucoidan was used in the following work.

### 2.4. Inhibition Against Activities of Adenosine Deaminase and Xanthine Oxidase in Liver of Mice Exposed to Adenine by Fucoidan

**Figure 1:**
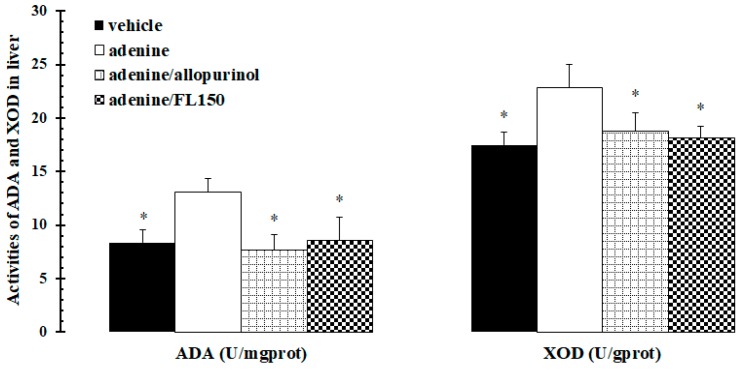
Fucoidan inhibited adenine-mediated increase of hepatic ADA and XOD activities of mice. Mice were treated with vehicle, adenine, adenine plus allopurinol, and adenine plus 150 mg/kg of fucoidan for 28 consecutive days. Activities of hepatic XOD and ADA in mice were determined with kits respectively according to manufacturer’s procedures. * *p* < 0.05 vs. adenine treated group.

Adenosine deaminase (ADA) and xanthine oxidase (XOD), two critical enzymes in purine nucleotide metabolism, convert purine to hypoxanthine and then to uric acid. Adenine administration promoted pronouncedly the activities of hepatic ADA and XOD, but an identical and dramatic fall of these activities occurred in both allopurinol and fucoidan exposed mice (Figure 1). The data suggested that fucoidan blocked urate production via inhibiting hepatic ADA and XOD.

### 2.5. Modification of Expression Profile of Renal Urate Transporter Gene in Adenine-Treated Mice

**Figure 2:**
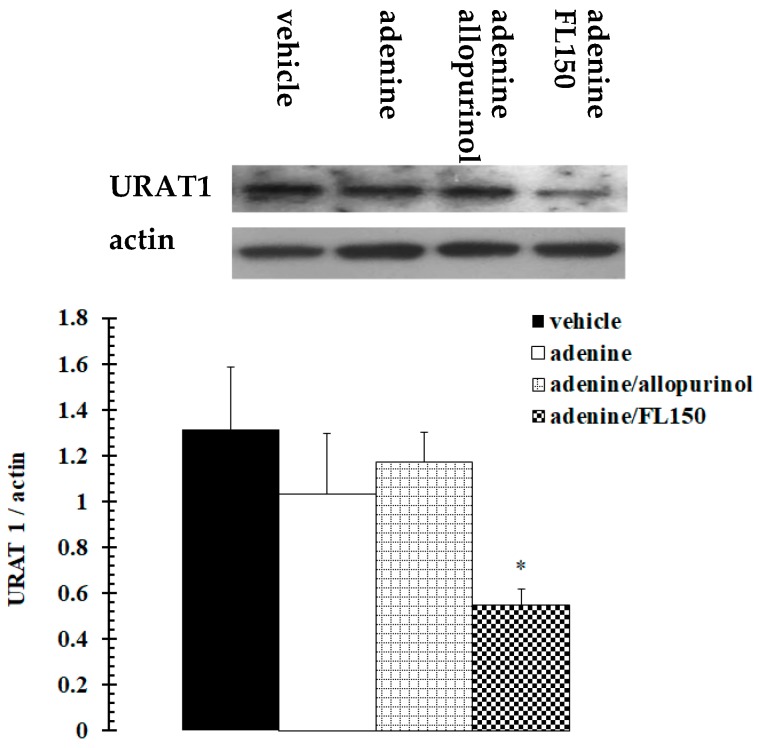
Fucoidan downregulated URAT1 expression in kidney of adenine-treated mice. Mice were treated with vehicle, adenine, adenine plus allopurinol, and adenine plus 150 mg/kg of fucoidan for 28 consecutive days. Content of URAT1 protein in kidney was assessed using assay of western blotting. URAT1 was observed at the site of 60 kDa; β-actin was observed at the site of 43 kDa. * *p* < 0.05 vs. adenine treated group.

**Figure 3:**
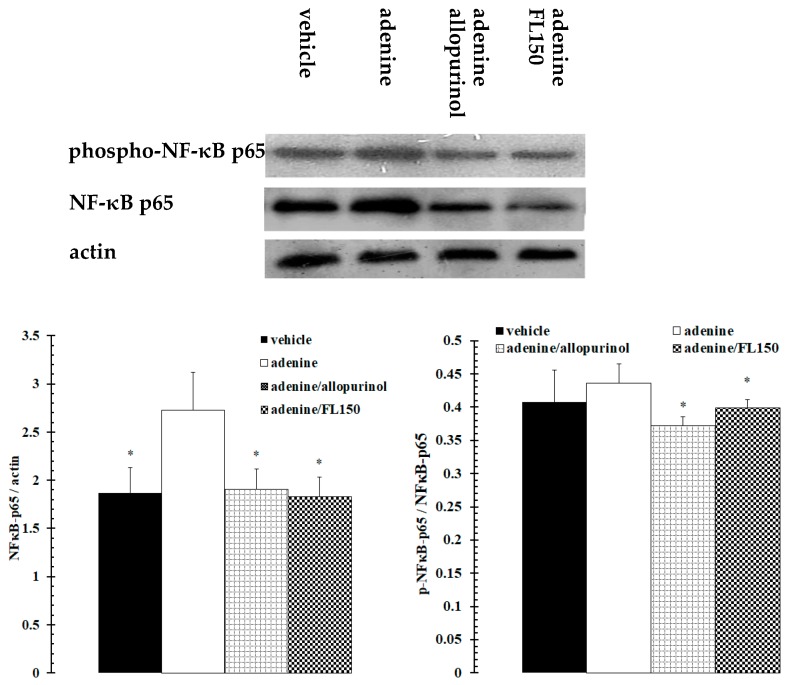
Fucoidan repressed adenine-induced expression and phosphorylation of NF-κB protein in kidney of mice. Mice were treated with vehicle, adenine, adenine plus allopurinol, and adenine plus 150 mg/kg of fucoidan for 28 consecutive days. Expression and phosphorylation of NF-κB protein was assessed using western blotting. NFκB p65, p-NFκB p65 (phospho-S536) and β-actin were observed at the sites of 64 kDa, 70 kDa and 43 kDa, respectvely. * *p* < 0.05 vs. adenine treated group.

Urate transporter 1 (URAT1) is one of the most important urate transporters which affect critical renal urate excretion. NF-κB is one of the important transcription factors involved. It was speculated using western blotting whether fucoidan regulated URAT1 and NF-κB signaling pathways involved in the regulatory mechanism. As Figure 2, the results showed that both adenine and allopurinol had no effect on the expression profile of the renal urate transporter gene, *urat1*, and there was also no visible difference in the expression profiles among vehicle, adenine and allopurinol treated mice. However, URAT1 was downregulated dramatically in fucoidan exposed mice. Meanwhile, the expression and activation of NF-κB p65 was promoted by exposure to adenine. Under administration of allopurinol or fucoidan, NF-κB p65 expression and activation were kept at normal levels (Figure 3).

## 3. Discussion

Hyperuricemia is a metabolic disease resulting from an imbalance between the endogenous production and renal excretion of uric acid, and correlates closely with hypertension, cardiovascular disease, insulin resistance, metabolic syndrome, diabetes mellitus, nonalcoholic fatty liver disease and renal disorders [6,7]. Due to imperfections of current clinical agents, natural products and Chinese traditional medicines are becoming important resources to screen for therapeutic drugs without side-effects for the treatment of hyperuricemia [6,7,8]. Fucoidan is a kind of natural sulfated polysaccharide from brown seaweed, and has been proven to have multiple bioactivities, including hepatoprotection [22,23,24] and renal protection [14,15,16,17,18,19].

It can be speculated from the literatures that fucoidan might be useful for treatment of hyperuricemia [14,15,16,17,22,23,24], however, that was first demonstrated by our current research. In our current work, adenine induced hyperuricemia and mediated hepatorenal dual disorders, were in fucoidan treated mice, prevented from causing adenine-mediated hepatic and renal damage. The involved mechanisms of uric acid mediated renal dysfunction include tubular toxicity, oxidative stress, inflammation, and so on [21,25]. Those might be critical factors for destroying the renal function of adenine-treated mice, demonstrated by alternations of the relative organ mass, uric acid, urea nitrogen, creatinine and antioxidative indicators. Fucoidan has been proven to have antioxidative activity [26,27], preventing uric acid mediated oxidative stress in the kidneys of mice and result in the recovery of renal function [15,16].

Similarly, high concentrations of uric acid also mediate hepatic toxicity. Reports showed that activities of alanine aminotransferase (ALT) and aspartate aminotransferase (AST) were modified in hyperuricemic rats that was hypoxanthine or potassium oxonate induced [28,29]. The hepatic damage was also observed in adenine induced hyperuricemic mice of the current research, which was attested by modifications to the relative organ mass, GOT and GPT activities and antioxidative capacity. Fucoidan administration blocked these modifications, which must be correlative to the antioxidative capacity of the marine sulfated polysaccharide. Hepatoprotection of fucoidan was also found in ethanol intoxicated rats, the increases in the activity of hepatic AST and ALT were inhibited by fucoidan, which caused the reduction of hepatic oxidative stress by the antioxidative effects of fucoidan [23]. Due to urate mediated disruption of hepatic function, alternations of hepatic ADA and XOD activities, two critical enzymes catalyzing the conversion of purine to uric acid [7], were impossible to escape. Our work firstly demonstrated that the activities of hepatic ADA and XOD in adenine mediated hyperuricemic mice were dramatically altered, and under the exposure of fucoidan, the activities of the enzymes were kept at normal levels, identical to vehicle treated mice, which was the reason of resulting in inhibition of uric acid production.

Apart from the inhibition of hepatic synthetic enzymes of uric acid, promotion of renal urate excretion is another critical therapeutic strategy for hyperuricemia. Kidneys account for 70% of excreted SUA, and approximately 90–95% of filtered uric acid is reabsorbed through proximal tubules [30,31]. Consequently SUA content depends on glomerular filtration and subsequent tubular reabsorption. Imbalance of the two processes causes hyperuricemia, less excretion resulting in primary hyperuricemia which accounts for about 90% of cases [32], has been considered as a marker for chronic kidney disease or an independent risk factor for the development of chronic kidney disease [33,34]. Two thirds of urate load is eliminated by kidneys on the basis of the synergistic effects of many urate transporters, such as URAT1 [20]. URAT1 functions in the reabsorption of urate [20]. Although adenine brought renal toxicity, it did not affect the expression of the transporter. Interestingly, the exposure to fucoidan not only avoided adenine-induced renal impairment, but also promoted renal excretion of urate, as demonstrated by the down-regulation of URAT1. These data indicated that fucoidan facilitated renal urate excretion and prevented hyperuricemia associated kidney toxicity.

It has been testified that NF-κB, a critical transcription factor that can activate a large number of pro-inflammatory genes, can be activated by uric acid [35], and that the activation can be inhibited by fucoidan via inhibition of NF-κB p65 subunit phosphorylation [17,36,37,38]. These results were re-showed by this research. This work revealed that activation of NF-κB p65 in mice exposed to allopurinol was inhibited, which may be caused by a uric acid decrease originating from the inhibition of allopurinol on ADA and XOD. In the kidneys of mice treated with FL, inhibition of NF-κB p65 protein phosphorylation was also observed. SUA reduction resulted from the combined action of FL on hepatic enzymes and renal URAT1 was a doubtless reason, while inhibition of NF-κB p65 protein activation by fucoidan should be another critical cause. Although there was no significant difference between allopurinol- and FL-exposed mice, the lower ratio of p-NF-κB p65/NF-κB p65 in allopurinol treated mice suggested that allopurinol exposure induced a stronger repression on the activation of the NF-κB p65 subunit. Data showed that both FL and allopurinol inhibited NF-κB p65 activation, but *urat1* gene expression was suppressed in FL exposed mice and unchanged in allopurinol treated mice, suggesting that URAT1 may be not a downstream target molecule of the NF-κB p65 signaling pathway. Presently, no evidence indicates a direct relationship between allopurinol and NF-κB p65 activation, or between NF-κB p65 signaling pathway and the *urat1* gene. Therefore it is impossible to deduce the involved regulatory mechanism, which is a critical issue that should be explained urgently. Currently, the therapeutic strategy for the treatment of hyperuricemia often aims to restrain acute episodes characterized by an inflammatory response of cells triggered by urate crystal deposition [6]. Therefore, fucoidan had an anti-inflammatory effect on hyperuricemia caused acute gout through suppressing activation of NF-κB signaling pathway. Meanwhile, fucoidan promotes renal urate excretion via modifying the expression profile of urate transporter URAT1.

Summarily, this work firstly testified the efficient treatment of fucoidan on hyperuricemia through two critical mechanisms, the promotion of renal urate excretion and the inhibition of hepatic urate production. Fucoidan represses the activities of hepatic ADA and XOD, resulting in a decrease of urate production, and downregulating URAT1 expression, leading to an increase in renal urate excretion. Although the NF-κB signaling pathway was regulated in kidneys of mice exposed to FL or allopurinol, it was impossible to determine whether the signaling cascade involved the regulatory mechanism of *urat1* gene expressional profile. Consequently, SUA content is reduced to normal levels. Moreover, adenine induced hepatorenal impairment was recovered by fucoidan. As a traditional Chinese medicine applied for more than one thousand years, *Laminaria japonica* is mainly grown in the coastal areas of China, Japan and Korea. FL is estimated to be approximately 1.66 g/100 g in dried *Laminaria japonica* and localized in an area between approximately 50–150 µm from the surface of *Laminaria japonica* [39]. Therefore, it can be concluded that fucoidan could be a potential therapeutic drug for treatment of hyperuricemia and acute gout because of its dual uricostatic and uricosuric effects.

## 4. Materials and Methods

### 4.1. Animals and Experimental Procedure

Male Kunming mice (20 ± 2 g) habituated in experimental conditions for 1 week were allocated to 6 groups, vehicle treated group, adenine treated group, allopurinol treated group (adenine plus allopurinol), and three fucoidan treated groups (adenine plus fucoidan), with 10 mice per group. Fucoidan was administered by gavage at dosages of 100, 150 and 200 mg/kg body mass every day, respectively, for 28 consecutive days. Dosages of adenine and allopurinol were 75 and 45 mg/kg body mass, respectively. Drug-delivery and administration time were the same as fucoidan. Animals were housed under standardized conditions in a room on a 12 h light/dark cycle with food and water available ad libitum. The current study was approved by the Animal Ethics Committee of Guangdong Ocean University, Zhanjiang, Guangdong, China.

### 4.2. Biochemistry Analysis

Blood was stored at 4 °C and allowed to coagulate to prepare serum. Liver and left kidneys were prepared in to a homogenate in normal saline. Following centrifugation, supernatants were collected. Biochemistry indicators of serum, liver and kidney were measured with detection kits according to the manufacturer’s protocol, including contents of uric acid, urea nitrogen, creatinine, MDA, and the activities of GOT, GPT, SOD and CAT. The kits were purchased from Nanjing Jiancheng Bioengineering Institute (Nanjing, China).

### 4.3. Western Blot Assay

Supernatant of renal homogenates in radio immuno precipitation assay lysis buffer containing phenylmethylsulfonyl fluoride (product of Beyotime institute of Biotechnology, Shanghai, China) were added with a sample buffer and denatured in boiling water for 5 min. After sodium dodecyl sulfate polyacrylamide gel electrophoresis, protein was transferred to a nitrocellulose membrane and then probed with a matched monoclonal antibody (antibodies of NF-κB p65 (ab16502), phosph-NFκB-p65 (ab86299) and URAT1 (ab76385) from Abcam, Cambridge, MA, USA; β-actin antibody (AF0003) from Beyotime Institute of Biotechnology, Shanghai, China) that could be captured by a horseradish peroxidase conjugated secondary antibody (product of Beyotime Institite of Biotechnology, Shanghai, China). A low background chemiluminesence detection system (W028-2, product of Beyotime institute of Biotechnology, Shanghai, China) was used to visualize the membrane with the target protein. β-actin was used as the internal reference. 

### 4.4. The Relative Organ Mass of Liver and Kidney

The relative organ mass ratio was calculated as liver or kidney mass (mg)/mouse body mass (g).

### 4.5. Data Statistical Analysis

Data were expressed as mean ± standard deviation, and analyzed by one-way analysis of variance using JPM software (7.0.2, SAS Institute Inc., Cary, NC, USA) and Tukey’s method. Statistical significance was set at 0.05 which were marked with an asterisk.

## Figures and Tables

**Table 1:** Fucoidan blocked adenine-induced changes of serum indicators of mice.

	Uric Acid (μmol/L)	Creatinine (μmol/L)	Urea Nitrogen (mmol/L)	GOT (U/gprot)	GPT (U/gprot)
vehicle	307.00 ± 56.61 *	74.83 ± 3.53 *	24.85 ± 1.17 *	67.81 ± 3.64 *	24.52 ± 2.3 *
adenine	737.22 ± 98.65	169.08 ± 6.61	42.11 ± 4.05	197.10 ± 16.56	78.87 ± 5.70
adenine/allopurinol	325.92 ± 43.46 *	79.99 ± 10.21 *	38.02 ± 3.21	65.68 ± 6.65 *	34.70 ± 3.31 *
adenine/FL100	373.66 ± 49.71 *	83.79 ± 8.68 *	26.96 ± 2.33 *	68.13 ± 8.50 *	29.53 ± 3.49 *
adenine/FL150	325.36 ± 67.46 *	76.76 ± 7.95 *	23.40 ± 2.40 *	67.75 ± 4.85 *	25.22 ± 2.05 *
adenine/FL200	368.41 ± 48.65 *	91.18 ± 8.74 *	25.73 ± 1.97 *	69.79 ± 10.48 *	30.53 ± 3.16 *

Notes: Mice were treated with normal saline (vehicle), adenine, adenine and allopurinol, adenine and FL (100, 150 or 200 mg/kg of fucoidan) for 28 consecutive days. Dosages of adenine and allopurinol were 75 and 45 mg/kg body mass, respectively. Asterisk (*) expresses *p* < 0.05, vs. adenine treated group.

**Table 2:** Effects of fucoidan on body weight and LRW of liver/kidney of mice exposed to adenine.

	Week 0	Week 1	Week 2	Week 3	Week 4		
	Body Weight (g)					Relative Weight (mg/g)	
						Liver	Kidney
vehicle	28.93 ± 2.69	36.75 ± 2.05 *	42.10 ± 1.73 *	43.10 ± 2.23 *	44.70 ± 2.95 *	5.38 ± 0.29 *	1.50 ± 0.18 *
adenine	28.57 ± 2.17	31.11 ± 1.91	29.56 ± 2.35	30.00 ± 3.42	30.28 ± 3.09	5.90 ± 0.39	2.16 ± 0.21
adenine/allopurinol	28.93 ± 1.31	31.44 ± 1.88	31.40 ± 4.14	34.16 ± 4.32	34.50 ± 3.13	4.99 ± 0.10 *	1.56 ± 0.21 *
adenine/FL100	28.96 ± 1.30	31.88 ± 1.40	33.07 ± 3.91	33.25 ± 3.15	31.95 ± 3.90	5.67 ± 0.22	1.77 ± 0.18 *
adenine/FL150	28.87 ± 1.35	32.18 ± 1.84	33.99 ± 2.76	34.67 ± 3.57	31.38 ± 3.25	5.08 ± 0.22 *	1.50 ± 0.10 *
adenine/FL200	28.63 ± 2.69	30.75 ± 2.05	33.81 ± 1.73	33.10 ± 2.23	32.70 ± 2.95	5.21 ± 0.20 *	1.52 ± 0.20 *

Notes: Mice were treated with normal saline (vehicle), adenine, adenine and allopurinol, adenine and FL (100, 150 or 200 mg/kg of fucoidan) for 28 consecutive days. Dosages of adenine and allopurinol were 75 and 45 mg/kg body mass, respectively. Asterisk (*) expresses *p* < 0.05, vs. adenine treated group.

**Table 3:** Fucoidan inhibited adenine mediated disruption of antioxidative capacity in liver or kidney of mice.

	SOD (U/gprot)		CAT (U/gprot)		MDA (nmol/100 mgprot)	
	Liver	Kidney	Liver	Kidney	Liver	Kidney
vehicle	211.20 ± 11.25 *	143.75 ± 7.60 *	17.96 ± 1.40 *	20.41 ± 1.53 *	69.00 ± 8.33 *	32.06 ± 4.03 *
adenine	143.31 ± 14.90	115.91 ± 6.33	12.72 ± 1.55	16.62 ± 1.21	114.03 ± 26.22	77.11 ± 6.05
adenine/allopurinol	153.12 ± 10.30	146.11 ± 23.37 *	14.90 ± 1.35	22.49 ± 1.61 *	67.10 ± 14.19 *	49.36 ± 7.09 *
adenine/FL100	199.74 ± 12.65 *	144.41 ± 16.09 *	14.03 ± 1.24	20.54 ± 1.38 *	81.04 ± 15.41 *	35.19 ± 9.01 *
adenine/FL150	218.51 ± 25.10 *	154.07 ± 15.53 *	17.81 ± 0.95 *	21.04 ± 1.57 *	62.16 ± 21.20 *	29.22 ± 3.16 *
adenine/FL200	170.01 ± 11.35	136.83 ± 7.73 *	13.78 ± 1.12	18.45 ± 2.41	89.23 ± 11.12	35.21 ± 12.01 *

Notes: Mice were treated with normal saline (vehicle), adenine, adenine and allopurinol, adenine and FL (100, 150 or 200 mg/kg of fucoidan) for 28 consecutive days. Dosages of adenine and allopurinol were 75 and 45 mg/kg body mass, respectively. Asterisk (*) expresses *p* < 0.05, vs. adenine treated group.
